# Diamond Blackfan Anemia at the Crossroad between Ribosome Biogenesis and Heme Metabolism

**DOI:** 10.1155/2010/790632

**Published:** 2010-05-05

**Authors:** Deborah Chiabrando, Emanuela Tolosano

**Affiliations:** Molecular Biotechnology Center, University of Torino, Via Nizza 52, 10126 Torino, Italy

## Abstract

Diamond-Blackfan anemia (DBA) is a rare, pure red-cell aplasia that presents during infancy. Approximately 40% of cases are associated with other congenital defects, particularly malformations of the upper limb or craniofacial region. Mutations in the gene coding for the ribosomal protein RPS19 have been identified in 25% of patients with DBA, with resulting impairment of 18S rRNA processing and 40S ribosomal subunit formation. Moreover, mutations in other ribosomal protein coding genes account for about 25% of other DBA cases. Recently, the analysis of mice from which the gene coding for the heme exporter Feline Leukemia Virus subgroup C Receptor (FLVCR1) is deleted suggested that this gene may be involved in the pathogenesis of DBA. FLVCR1-null mice show a phenotype resembling that of DBA patients, including erythroid failure and malformations. Interestingly, some DBA patients have disease linkage to chromosome 1q31, where FLVCR1 is mapped. Moreover, it has been reported that cells from DBA patients express alternatively spliced isoforms of FLVCR1 which encode non-functional proteins. Herein, we review the known roles of RPS19 and FLVCR1 in ribosome function and heme metabolism respectively, and discuss how the deficiency of a ribosomal protein or of a heme exporter may result in the same phenotype.

## 1. Introduction

Diamond Blackfan anemia (DBA; OMIM #205900) is a rare, congenital, pure red-cell aplasia that presents during infancy, usually within the first year of life. Its main clinical features are normochromic and macrocytic anemia, reticulocytopenia, and nearly complete absence of red blood cell precursors in the bone marrow. The erythroid hypoplasia is due to impaired proliferation and differentiation of red blood cell progenitors in the bone marrow. Other hematopoietic lineages are usually normal. High serum levels of folic acid, vitamin B12, erythropoietin, elevated fetal hemoglobin, and erythrocyte adenosine deaminase further support DBA diagnosis [1]. 

 DBA is a clinically heterogeneous disorder. In addition to erythroid failure, it is also characterized by congenital malformations and cancer predisposition. Growth retardation and a wide variety of congenital anomalies have been seen in more than 50% of DBA cases. Short stature is constitutional in most patients. Thumbs, upper limbs, and hands and craniofacial, urogenital, and cardiovascular anomalies are also common. Although not yet statistically validated, DBA patients have an increased risk of cancer development. Both hematopoietic malignancy (acute myeloid leukemia, myelodysplastic syndrome, Hodgkin and non/Hodgkin lymphomas, and acute lymphoblastic leukemia) and nonhematopoietic tumors (osteogenic sarcoma, breast cancer, hepatocellular carcinoma, melanoma, fibrohistiocytoma, gastric cancer, and colon cancer) have been described in some DBA patients [2, 3].

 DBA is an autosomal dominant disorder with a disease incidence of 5–10 cases per million live births in Europe. The molecular genetics of DBA is evident in about half of the patients. All the DBA mutations identified so far, both in sporadic and familial cases, have been found in genes coding for ribosomal proteins, and DBA is now considered the prototype of “ribosome-based disorders”. The first DBA gene to be identified is Ribosomal Protein S19 (RPS19), located on chromosome 19q13.2 and mutated in 25% of DBA patients. Recently, mutations in several other ribosomal proteins, RPS24, RPS17, RPL11, RPL5, RPS7, and RPL35a, have been identified in approximately 20% of DBA patients [4]. In the remaining 55% of DBA patients, no mutations have been reported suggesting the existence of other genes involved in the pathogenesis of DBA.

 Recently, it has been demonstrated that Feline Leukemia Virus subgroup C Receptor (FLVCR1) deficient embryos display a phenotype very close to DBA patients, including bone marrow failure and congenital malformations [5]. FLVCR1 is a heme exporter [6, 7] suggesting that altered heme homeostasis could play a fundamental role in the pathogenesis of DBA. Although no mutations in FLVCR1 have been found in DBA patients [8], it will be interesting to understand how the deficiency in a heme exporter could recapitulate the human disease and if a link between FLVCR1 and the ribosome biosynthetic pathway exists. In this paper, the possible molecular mechanisms underlying the pathogenesis of DBA are discussed.

## 2. Diamond-Blackfan Anemia as a Disorder of Ribosome Biogenesis

### 2.1. Ribosomal Protein S19

RPS19 is one of 33 ribosomal proteins that, together with 18S rRNA, constitute the 40S ribosomal subunit. Immunoelectron microscope studies showed that it localizes to the external surface of the 40S subunit, in close proximity to RPS3A, RPS13, RPS16, and RPS24, a region that interacts with the eukaryotic initiation factor eIF-2 [9]. 

 RPS19 is a highly conserved, ubiquitously expressed protein. In red blood cells, RPS19 expression strongly decreases during terminal erythroid differentiation [10]. RPS19 is localized predominantly in the nucleus, in particular into the nucleolus where ribosome synthesis takes place, and in the cytoplasm as a ribosomal component [11].

 Different kinds of mutation in RPS19 have been discovered in DBA patients: nonsense and missense mutations, deletions, insertions, splice defects, and larger rearrangements. RPS19 mutations could lead to reduction of mRNA and/or protein levels, loss of nucleolar localization, or impairment of ribosomal association [12]. All the mutations identified so far in ribosomal proteins have been found in heterozygosity. How the haploinsufficiency of a ribosomal protein may lead to erythroid failure, malformations, and cancer is still not completely understood and the multiple molecular mechanisms in which ribosomal proteins are involved are only just becoming clearer.

### 2.2. In Vitro and In Vivo Models of RPS19 Deficiency

The erythroid hypoplasia characterizing DBA is due to a defect of erythroid progenitor differentiation. CD34+ bone marrow cells derived from DBA patients and cultured in vitro show a reduction in proliferation rates and colony formation capacity associated with increased apoptosis [13, 14]. 

 The fact that RPS19 has a fundamental role during erythropoiesis was first evident when RPS19 mutations were identified in DBA patients. This role was further confirmed by in vitro studies in which the effect of RPS19 silencing and overexpression was analyzed. In both primary culture (CD34+ umbilical cord blood and bone marrow cells) and erythroid-like cell lines (TF-1 and U-7 cells) RPS19 silencing mimics the DBA phenotype: impaired erythroid differentiation and proliferation of erythroid progenitors, cell growth arrest at G0/G1, and apoptosis [13, 15]. Similar results were obtained by overexpressing mutated forms of RPS19, analogous to those found in DBA patients, in K562 cells and in human CD34+ bone marrow cells [16]. On the contrary, RPS19 overexpression into CD34+ bone marrow cells from RPS19-deficient DBA patients increases the erythroid colony formation capacity and improves erythroid progenitors proliferation in vitro [17–19]. Furthermore, the loss of different ribosomal proteins causes different cell cycle defects; primary fibroblast from DBA patients with RPS19 mutations is characterized by cell cycle arrest at the G1 phase while RPS24 mutation impairs the progression through the S phase [20]. 

 Generation of a mouse model of RPS19 deficiency has been attempted by Matsson and colleagues but the deletion of RPS19 in mice is lethal before implantation; no RPS19 deficient embryo may be recovered as early as the blastocyst stage. Moreover, RPS19 heterozygous mice are viable but indistinguishable from wild-type mice: hematological parameters, erythroid proliferation, and differentiation are normal as well as organ development and morphology. No difference may be found in DBA markers such as erythrocyte adenosine deaminase and globin isoforms. Contrary to the occurrence in human patients, heterozygous mice fully compensate the loss of one RPS19 allele with normal protein levels [21, 22].

On the other hand, zebrafish appears to be a good animal model to study the consequences of RPS19 deficiency. Knocking-down RPS19 in zebrafish, using antisense morpholinos, perfectly recapitulates the DBA phenotype. Both the primitive and definitive waves of erythropoiesis are impaired: an increased number of red cell progenitors remains in the intermediate cell mass and primitive cells are not replaced by adult ones. Also erythroid markers of differentiation, such as gata-1 and c-myb, are expressed at lower levels compared to controls. Myeloid and lymphoid lineages appear normal. Morphants also show delayed development and morphological defects that are evident during somatogenesis: reduced forebrain and eye field, smaller head and eyes with head cartilage not formed properly [23–25]. Similar phenotypes are observed when the expression of other ribosomal proteins is lost. The knock-down of RPL35, RPL35a, and RPLP2 in zebrafish leads to erythroid failure and malformations [24], while the loss of RPL11 only leads to alteration of embryonic development [26]. These data highlight an important role for ribosomal proteins not only during erythropoiesis but also for proper organ development. 

 The fundamental role of ribosome integrity for erythropoiesis and development is further confirmed by the observation that a heterogeneous group of disorders, referred to as “inherited bone marrow failure syndromes”, is also characterized by bone marrow failure, congenital malformations, and cancer predisposition. In addition to DBA, this group of disorders include dyskeratosis congenita (DC), cartilage-hair hypoplasia (CHH), and Shwachman-Diamond syndrome (SDS). In each case, a defect in proteins that are involved in ribosome synthesis has been identified. DC is due to mutations in dyskerin gene (DKC1), a putative pseudouridyl synthase involved in rRNA modification. Mutations in RMPR, which encode the RNA component of ribonuclease mitochondrial RNA processing complex (RNase MRP), involved in the cleavage of rRNA precursors and other functions, have been identified in CHH. SDS is due to mutations in SBDS, which encode for a protein involved in ribosome synthesis [1].

### 2.3. Role of RPS19 in Ribosome Biogenesis

The mammalian ribosome is composed of a large 60S and a small 40S subunit consisting of 4 rRNAs and 80 ribosomal proteins. Ribosome biogenesis is a very complex process. First, a polycistronic pre-rRNA transcript is processed into the 18S rRNA (component of the 40S subunit) and the 5.8S and 25S/28S rRNAs (component of the 60S subunit). Then, rRNAs associate with the ribosomal proteins and the 5S rRNA, which is independently transcribed, and form an early 90S particle that is subsequently processed into 66S and 43S preribosomes. Following assembly in the nucleolus, the preribosomes are exported into the cytoplasm through the nuclear pore complex as independent entities and additional maturation steps are necessary to achieve translational competence. RPS19 is one of the ribosomal proteins that, together with 18S rRNA, constitute the small 40S subunit [27]. 

Recent studies demonstrated that RPS19 not only has a structural role, as initially thought, but also has additional functions. The analysis of the RPS19 interactome, by a high throughput proteomic approach, suggests that RPS19 may play an important role in rRNA processing, metabolism, and translation. One hundred and fifty-nine new proteins interacting with RPS19 have been identified: NT-Pases, hydrolases/helicases, isomerases, kinases, splicing factors, structural constituents of ribosomes, transcription factors, transferases, transporters, and DNA/RNA-binding protein. It has been demonstrated that RPS19 not only interacts with other components of the 40S ribosome but also with the preribosome 90S. Moreover, RPS19 interacts with proteins important for the transport of the small subunit from the nucleus to the cytoplasm and also with proteins involved in the pseudouridylation of rRNA, a process involved in early stages of ribosome biogenesis [28]. 

 Interference with RPS19 expression in vitro leads to a reduced maturation of the 40S ribosome subunit, increased expression of the 60S subunit, reduced 18S rRNA synthesis, and accumulation of a novel 21S pre-rRNA [1, 29]. The same results were obtained analyzing bone marrow CD34+ cells and fibroblasts from DBA patients [13, 30]. These data suggest that RPS19 might be required for a specific step in 18S rRNA processing from the 21S precursor, highlighting that RPS19 actively participates in ribosome biogenesis.

 Recent works showed that RPS19 haploinsufficiency leads to the posttranscriptional downregulation of other small subunit ribosomal proteins such as RPS20, RPS21 and RPS24 compared to large subunit ribosomal proteins. This has been demonstrated in TF1 cells interfered for RPS19 and in lymphoblastoid and fibroblast cell lines derived from RPS19 deficient DBA patients [20, 31] suggesting the importance of the correct subunits amount and stoichiometry for ribosome assembly.

### 2.4. Role of p53 Activation in the Pathogenesis of DBA

Recent data suggest an important role of p53 family in the pathogenesis of DBA. The knock-down of RPS19 in zebrafish leads to erythroid and developmental defects which are associated with over-expression of p53 and ΔNp63 [23]. The p53 family plays a crucial role in cell proliferation and differentiation during development and p53 activation promotes cell growth arrest and apoptosis, while ΔNp63 over-expression supports proliferation. It has been hypothesized that an imbalance between p53 and ΔNp63 may affect the ability of red cell precursors to differentiate. Moreover, ΔNp63 has a crucial role in specification of nonneural ectoderm during gastrulation, thus likely contributing to the craniofacial defects seen in morphants. The phenotype of RPS19 deficient embryos is alleviated when the expression of p53 and ΔNp63 is down-regulated [23].

 Similar results have been observed when the expression of RPL11 is knocked-down in zebrafish. Also the loss of RPL11 leads to developmental defects associated with the activation of p53 pathway [26]. 

 Interestingly, the activation of p53 has been observed also when the expression of other ribosomal proteins not directly involved in the pathogenesis of DBA, like RPS8, RPS11, RPS18, and RPS6, is lost [23, 32], or when the expression of other factors involved in ribosome biogenesis like Bop1 [33], bap28 [34], or TCOF is impaired [35]. These data suggest that impairment of ribosome biogenesis in general leads to the activation of p53 pathway.

 Disruption of the nucleolus, the region where ribosome biogenesis takes place, also leads to p53 activation [36]. Therefore it has been hypothesized that ribosome biogenesis impairment due to mutations in RPS19 could lead to nucleolar stress, p53 activation, erythroid failure, and malformation typical of DBA.

### 2.5. Role of RPS19 during Erythropoiesis

How the haploinsufficiency of a ribosomal protein could lead to erythroid impairment is still an open question. It has been hypothesized that immature erythroid progenitors could be particularly sensitive to ribosomal proteins deficiency with respect to other cell types because of their increased proliferation rates. High rates of RNA synthesis which exceeded the cell proliferation rates have been shown in primary erythroid culture [37]. So these cells could be more sensitive to ribosome biogenesis impairment due to RPS19 loss than other cell types. This also correlates with the high expression of RPS19 in the early phases of erythropoiesis [10, 38].

 Furthermore, Sieff and coworker showed for the first time that loss of RPS19 specifically affects erythropoiesis as the knock-down of RPS19 in primary murine fetal liver erythroid cells results in the downregulation of key erythroid signaling proteins [37]. Loss of RPS19 decreases the expression of MYB, a transcription factor critically important for erythropoiesis, and of its transcriptional target KIT. The downregulation of MYB has also been shown using global gene expression analysis on bone marrow progenitors from DBA patients [37].

 So impairment of ribosome biogenesis during the early stages of erythropoiesis leads to the deficiency of critically important erythroid transcription factors.

## 3. Diamond Blackfan Anemia as a Putative Disorder of Heme Metabolism

### 3.1. Feline Leukemia Virus Subgroup C Receptor

Feline Leukemia Virus subgroup C Receptor (FLVCR1) is a heme exporter belonging to the major facilitator superfamily (MFS) of transporters [39]. Overexpression of FLVCR1 in the rat renal epithelial cell line NRK causes a slight decrease in intracellular heme concentration while the impairment of FLVCR1 expression in feline embryonic fibroblasts leads to increased intracellular heme levels. NRK cells overexpressing FLVCR1 show an increase in heme export measured using both the fluorescent heme analog zinc mesoporphyrin and Fe^59^-hemin [7].

 FLVCR1 is ubiquitously expressed [5]. High levels of FLVCR1 expression have been detected in Caco-2 and HepG2 cell lines, cancer cell lines, peripheral blood CD34+ stem cell progenitors, and hematopoietic cell lines with erythroid features [7].

### 3.2. Role of FLVCR1 during Erythropoiesis

FLVCR1 has been initially identified as the receptor for Feline Leukemia Virus subgroup C (FeLV C) causing a severe erythroid aplasia in cats similar to the anemia seen in DBA patients, suggesting that FLVCR1 plays a fundamental role during erythropoiesis [40]. 

 FLVCR1 expression is high in early erythroid precursors and decreases during erythroid differentiation, similar to RPS19 expression. Cell lines with undifferentiated erythroid features like K562 or HEL-DR express high levels of FLVCR1 while it is expressed at low levels in HEL-D, a more mature cell line with spontaneous hemoglobinization. In the same way, FLVCR1 is expressed at high levels in CFU-E from mobilized peripheral blood CD34+ stem progenitor cells and its expression decreases during erythroid differentiation in vitro. Impairment of FLVCR1 function, both in K562 cells and in lineage-depleted human umbilical cord blood cells, results in a decreased erythroid differentiation and increased apoptosis [7]. In vivo, the loss of FLVCR1 in mice results in embryonic lethality due to the impairment of definitive erythropoiesis. Also when FLVCR1 expression is deleted postnatally, mice develop severe anemia [5].

### 3.3. Erythropoiesis and Heme Homeostasis

Heme is a complex of iron and protophorpyrin IX fundamental for cell biology. Heme is the prosthetic group of many essential proteins including hemoglobin, myoglobin, and cytochromes. However, high concentrations of intracellular free heme are toxic, causing cell oxidative damage through lipid peroxidation. So the balance between heme biosynthesis, heme utilization for hemoprotein production, and heme catabolism is controlled at multiple levels [41].

**Figure 1:**
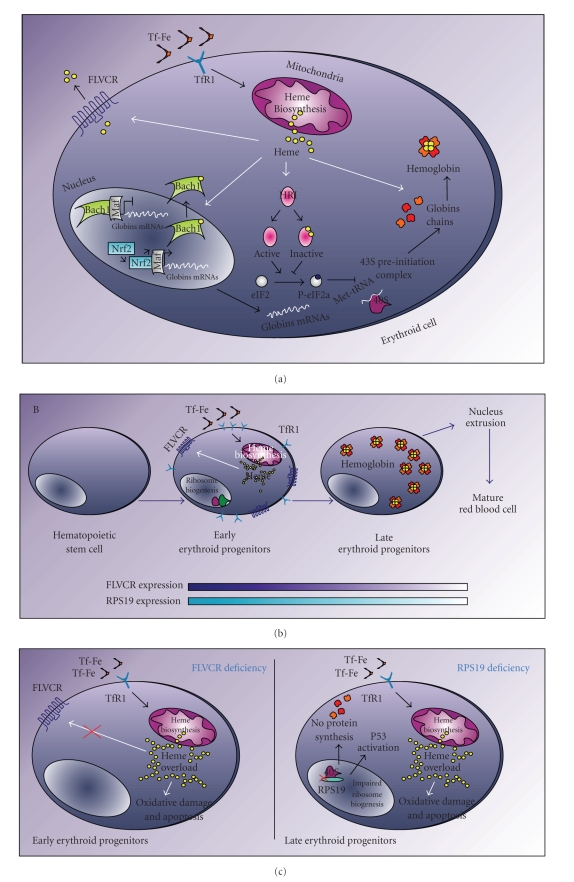
(a) Multiple roles of heme during erythroid cell differentiation. (b) Role of FLVCR1 during erythroid differentiation. (c) A model to explain how both FLVCR1, and RPS19 deficiencies may result in the failure of erythroid differentiation.

 Moreover, heme is not only a structural component of hemoproteins but it is also involved in many intracellular pathways. Intracellular heme regulates both the transcription and translation of globin chains through interaction with the transcriptional repressor Bach1 and the heme-regulated eIF2*α* kinase (HRI), respectively (Figure 1(a)).

 Heme positively regulates the transcription of globin mRNAs through binding to the transcriptional repressor Btb and Cnc Homology 1 (Bach1). Under basal condition, Bach1 heterodimerizes with small Maf transcription factors and binds to MARE (Maf recognition elements) sequences inhibiting the transcription of target mRNAs. Heme binding to Bach1 decreases its affinity to small Maf proteins and promotes Bach1 nuclear export; small Maf proteins heterodimerize with Nuclear Factor Erythroid 2-like (Nrf2) proteins activating the transcription of target genes [42]. So, when intracellular heme concentration increases, the transcription of globin mRNAs increases.

 HRI is a kinase able to phosphorylate the translation initiation factor eIF2*α* in a heme-dependent manner. Phosphorylation of eIF2 leads to inhibition of protein synthesis. During erythropoiesis when intracellular heme increases, heme binding to HRI keeps HRI in an inactive state, thus allowing protein synthesis. During this stage of erythroid differentiation, the main proteins to be synthesized are *α*- and *β*-globin chains and so hemoglobin is formed [43]. The regulation of HRI activity by intracellular heme allows for coordination of the synthesis of globin chains with heme concentration. Imbalance between heme and globin chains is deleterious to red blood cells and their progenitors as intracellular free heme promotes oxidative cell damage and apoptosis. It has been hypothesized that FLVCR1 could represent an additional control step in this process [7]. Because FLVCR1 expression is high during the earlier stages of erythropoiesis, it has been suggested that FLVCR1 exports excess heme out of erythroid progenitors when globin synthesis is not yet initiated. Then, as erythroid differentiation proceeds, globin chains synthesis initiates, FLVCR1 expression decreases, and intracellular heme concentration increases allowing hemoglobin formation (Figure 1(b)). According to this model, the loss of FLVCR1 expression leads to increased intracellular free heme, cytotoxicity, and impaired differentiation (Figure 1(c)).

### 3.4. Role of FLVCR1 during Development

In addition to the erythroid defect, FLVCR1-null embryos display defective growth and developmental anomalies resembling those found in DBA [5]: abnormal limb, hand, and digit maturation, flattened faces, and hypertelorism. These data suggest that FLVCR1 expression is also fundamental for proper organ development. 

 It has been observed that hemoglobin, heme, and bilirubin have an inhibitory effect on cartilage metabolism and growth in vitro although the molecular mechanism is not clear [44–46]. FLVCR1 is expressed at high levels during development in the yolk sac and placenta [5]. It is possible that beyond a role in erythropoiesis, FLVCR1 could be also involved in maternal-fetus heme exchange. If so, we can hypothesize that the lack of FLVCR1 during development may determine a heme overload condition in the embryo and, consequently, an inhibition of cartilage development, resulting in malformations.

### 3.5. Links between FLVCR1 and DBA

It is now clear that DBA is a clinically heterogeneous disorder and no clear genotype-phenotype correlation has been found. Congenital malformation is constitutional in most patients, but the type and degree of severity of these anomalies is also different among DBA patients affected by the same mutation. In the same way there is no correlation between genotype and cancer predisposition or response to therapies. Mutations in ribosomal proteins account for about half of DBA cases, but the molecular genetics of the remaining cases is still unknown, suggesting the existence of other DBA genes or unidentified modifiers that modulate the phenotype of DBA.

 Interestingly, FLVCR1-null mice display a phenotype very close to DBA. Impairment of erythropoiesis occurs at the same stage as that seen in DBA patients, and also the kind of malformations seen in FLVCR1-null mice are very similar to the congenital abnormalities described in DBA patients. 

 Human FLVCR1 has been mapped by radiation hybrid mapping to chromosome 1q31.3. Apart from the transcription factor ATF3, no other genes have been mapped to this chromosome region. It is intriguing that rearrangement of the distal region on chromosome 1q has been described in a patient with DBA. Mutations in FLVCR1 have been searched in a small group of multiplex DBA families with disease linkage to 1q31 but no mutations have been identified [8]. 

Alternatively spliced isoforms of FLVCR1 have been identified in immature bone marrow erythroid cells of some DBA patients negative for RPS19 gene mutations. Four different FLVCR1 isoforms result from the deletion of exon 2 or exon 3 alone or in combination with exon 6 [47]. 

 Splicing of FLVCR1 exon 3 or exon 6 causes in-frame mutations that encode potential proteins with major deletions. Splicing of exon 2 causes a frame-shift mutation resulting in a premature stop codon and potentially encodes a truncated isoform of the protein.

 In vitro, overexpression of deleted exon 3 or exon 6 FLVCR1 isoforms leads to phenotypes that are weakly susceptible to FeLV-C infection, indicating that FLVCR1 function is impaired. These isoforms are expressed at lower levels than normal FLVCR1 and are mislocalized: while canonical FLVCR1 is expressed at the cell membrane, alternative spliced FLVCR1 isoforms localize intracellularly. Moreover, immature erythroid cells from DBA patients show a decreased expression of FLVCR1 mRNA and an enhancement of alternative spliced transcripts. It is interesting that RPS19 interference in K562 cells results in an increase of FLVCR1 alternative splicing, establishing the first link between RPS19 and FLVCR1 in the pathogenesis of DBA [47].

## 4. Conclusions

DBA is a clinically heterogeneous disorder characterized by hypoplastic anemia associated with congenital malformations, and cancer predisposition. The molecular mechanisms underlying the pathogenesis of DBA are still not completely understood. Mutations in RPS19 cause an impairment of ribosome biogenesis and protein synthesis but how this leads to erythroid failure, congenital malformations and cancer is unknown. It has been hypothesized that impairment of ribosome biogenesis leads to nucleolar disorganization and activation of the p53 family.

 It is intriguing to note the similarity between the phenotype of FLVCR1-null embryos and DBA. It has been hypothesized that the loss of both FLVCR1 and RPS19 could converge on the same pathway. Loss of FLVCR1 causes increased intracellular heme levels during a stage of erythroid differentiation in which globin chains synthesis is not yet initiated. The haploinsufficiency of RPS19 leads to impaired ribosome biogenesis and therefore protein synthesis. As globin chains are the major proteins synthesized during erythroid differentiation, there is an increase in intracellular free heme. In both cases, high intracellular heme concentration would result in the block of erythroid differentiation and apoptosis (Figure 1(c)). This hypothesis clearly explains the erythroid phenotype but not how RPS19 or FLVCR1 deficiency leads to congenital malformations. As mentioned earlier, heme is a potent inhibitor of cartilage growth and metabolism. Thus, even in this case it is possible to speculate that heme overload resulting from FLVCR1 gene function impairment and reduced protein synthesis due to ribosomal protein genes mutations may generate the same phenotype, that is, skeletal malformation. Interestingly, it has been reported that perichondrial zone expresses high levels of genes involved in protein synthesis including RPL35a and RPS6, as well as the hemoglobin beta2 gene.

 Finally, the work of Rey and colleagues suggested a direct relationship between RPS19 and FLVCR1 [47]. Interference with RPS19 results in a decreased expression of FLVCR1 and in an increased level of alternative spliced isoforms of FLVCR1, with compromised function. It could be speculated that RPS19 is able to directly or indirectly regulate the expression of FLVCR1 but the molecular details of this relationship are yet to be understood. Moreover, nothing is known about the proteins interacting with FLVCR1 and so we cannot exclude the fact that RPS19 and FLVCR1 may share common downstream interactors.

 The availability of cellular and animal models for studying ribosomal proteins and FLVCR1 gene function constitutes the basis for future work aimed at elucidating the molecular pathogenetic mechanism of DBA.
